# Effects of COVID-19-Related Anxiety and Sleep Problems on Loneliness and Feelings of School Refusal in Adolescents

**DOI:** 10.3389/fpsyt.2022.918417

**Published:** 2022-06-14

**Authors:** Isa Okajima, Yukako Honda, Osamu Semba, Yoji Kiyota, Yasuo Tani

**Affiliations:** ^1^Behavioral Sleep Medicine and Sciences Laboratory, Department of Psychological Counseling, Faculty of Humanities, Tokyo Kasei University, Tokyo, Japan; ^2^Graduate School of Human Life Sciences, Tokyo Kasei University, Tokyo, Japan; ^3^BiosPyxis Co., Ltd., Tokyo, Japan; ^4^Medimpl Corporation, Tokyo, Japan; ^5^Inochi and Future Strategy Headquarters Office, Policy Bureau, Kanagawa Prefectural Government, Kanagawa, Japan

**Keywords:** chronotype, COVID-19, insomnia, loneliness, school refusal, sleep debt

## Abstract

**Background:**

COVID-19-related anxiety, sleep problems, and loneliness may be risk factors for school refusal in children and adolescents. However, few studies have examined the mechanisms by which these risk factors cause school refusal. This study examined the process by which COVID-19-related anxiety, sleep problems, and loneliness cause school refusal, using structural equation modeling.

**Methods:**

In this cross-sectional questionnaire-based study, 256 (109 male, 147 female, mean age: 15.37 ± 0.48 years) senior high school students were asked to complete the Stress and Anxiety associated with Viral Epidemics-6 questionnaire to assess COVID-19-related anxiety, the Athens Insomnia Scale (AIS), Sleep Debt Index (SDI), and chronotype (MSFsc) to assess sleep problems, the Three-Item Loneliness Scale (TILS) to assess loneliness, and Feelings of School-Avoidance Scale (FSAS) to assess school refusal.

**Results:**

Structural equation modeling showed that sleep problems affected loneliness (β = 0.52) and feelings of school refusal (β = 0.37), and that loneliness affected feelings of school refusal (β = 0.47). There was no significant pathway of COVID-19-related anxiety on sleep problems, loneliness, or feelings of school refusal. The indirect effect of sleep problems on feelings of school refusal through loneliness was significant. The results of hierarchical multiple regression analysis showed that AIS (β = 0.30) and SDI (β = 0.13) scores were associated with TILS, and AIS (β = 0.26) and MSFsc (β = −0.14) scores were associated with FSAS scores.

**Conclusion:**

The findings of this study showed that sleep problems affected feelings of school refusal *via* both direct and indirect pathways through the exacerbation of loneliness. To prevent school refusal in adolescents, addressing the indirect pathway *via* loneliness could be effective in improving insomnia and sleep debt, while addressing the direct pathway could be effective in improving insomnia and chronotype.

## Introduction

Continued absence from school, that is, school refusal, is a serious problem that has significant short- and long-term consequences, such as worsening of peer relationships, poorer academic achievement (1, 2), early school leaving, and later unemployment and health problems (3–5).

In Japan, prolonged school absenteeism (≥30 days per year), excluding those caused by illness and financial reasons, is defined as school refusal. The number of Japanese children with school refusal has increased over eight consecutive years (6). In 2020, it was reported that the number of students per 1,000 students in elementary school, junior high school, and senior high school who demonstrated school refusal was 10, 41, and 14, respectively. However, the rate of return to school has been decreasing over the years. Over 98% of Japanese children go to senior high school (6), and graduation from senior high school has become almost the minimum requirement for a young Japanese person to obtain employment (7). Therefore, school refusal tends to hinder career options and social independence.

Several issues, such as anxiety, loneliness, and sleep problems, are associated with school refusal. It has been reported that children with school refusal may suffer from significant overt anxiety symptoms such as fears of separation, tests or teachers, or transition (5). Recently, approximately 12% of Japanese students avoided going to school due to anxiety about the coronavirus disease 2019 (COVID-19) infection (COVID-19-related anxiety) after COVID-19 pandemic (6). Loneliness is also a factor associated with school refusal. A previous multi-regression analysis showed that loneliness was associated with feelings of school refusal (β = 0.24) among adolescents (8).

Of sleep problems, insomnia, chronotype (an individual's preference for sleep timing), and sleep debt (chronic short sleep duration due to sleep restriction or sleep deprivation) are associated with school refusal (9–11). For example, children suffering from insomnia (sleep onset problems, difficulties maintaining sleep) showed significantly higher scores in school refusal behavior than did children without sleep problems (9). In addition, expanded sleep duration (i.e., elimination of sleep debt) through sleep education may lead to a reduction in the number of children with school absenteeism (11).

The relationships among anxiety, loneliness, and sleep problems have been also reported in previous studies (12–14). A systematic review and meta-analysis showed that loneliness is correlated with self-reported sleep problems (12). Hom et al. (15) conducted six studies on the relationship between sleep problems and loneliness. These studies showed that the bivariate association between insomnia and loneliness was significant and moderate in magnitude, and that these relationships were stronger in younger participants (15). Furthermore, Lack of sleep leads to a behavioral profile of social withdrawal and loneliness (16), and insomnia affects suicidal ideation *via* loneliness (17).

In addition, an association between sleep problems and loneliness was shown to be strong among individuals with more COVID-19-related anxiety (13). Recently, it was revealed that COVID-19-related anxiety contributes to increased insomnia, depression, and anxiety (14, 18). In previous studies before COVID-19 pandemic, sleep debt and insomnia were associated with anxiety symptoms, but chronotype was not (19–21).

**Figure 1:**
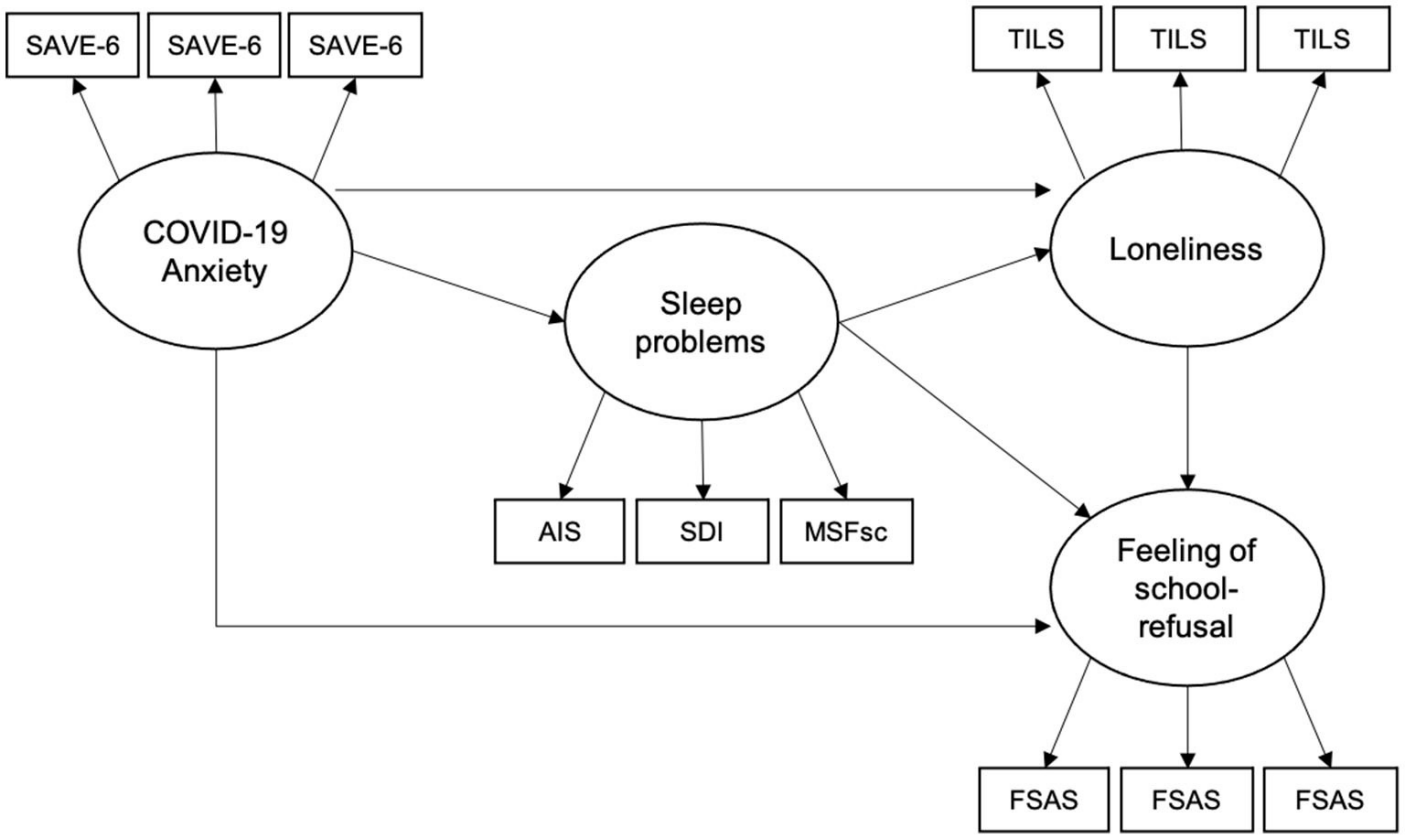
Structural equation modeling of hypothesized model.

Thus, COVID-19-related anxiety, sleep problems, and a higher level of loneliness may be risk factors for school refusal among children and adolescents. However, few studies have examined the mechanisms by which these risk factors cause school refusal. This study aimed to examine the following process model using structural equation modeling (SEM; Figure 1): COVID-19-related anxiety affects sleep problems, loneliness, and feelings of school refusal; sleep problems affect loneliness and feelings of school refusal; and loneliness affects feelings of school refusal.

## Materials and Methods

This study was approved by the Ethics Committee of Tokyo Kasei University (ID: Ita-E2021-15). All study participants provided their informed consent to take part in this study, in accordance with the Declaration of Helsinki.

### Participants

The data analyzed in the in-person-survey were collected from July to August 2021. The study sample comprised 277 students from a public senior high school in Kanagawa Prefecture. Of these students, 256 (109 male, 147 female, mean age: 15.37 ± 0.48 years) students who had completed the following measures and who had a sleep debt index (SDI) score of zero or more (22) were selected and analyzed.

### Measures

#### Demographic Data

The participants were asked to provide their age and sex.

#### COVID-19-Related Anxiety

The Stress and Anxiety associated with Viral Epidemics-6 (SAVE-6) is a validated 6-item self-report questionnaire that assesses anxiety and distress in response to viral epidemics (14, 23). The scores for the SAVE-6 in relation to the COVID-19 pandemic were summed, with higher scores indicating greater anxiety and distress.

#### Sleep Problems

To measure sleep problems, we used the Athens Insomnia Scale (AIS) for insomnia, the sleep-corrected Midpoint of Sleep on Free days (MSFsc) for evaluating chronotype, and the Sleep Debt Index (SDI) to assess sleep debt.

The AIS is a validated 8-item self-report questionnaire that assesses insomnia severity (24–26). The AIS score was summed, with higher scores indicating more severe insomnia. A cut-off score of 5.5 points for AIS was previously determined; therefore, in the present study, respondents with AIS scores of ≥6 were considered to have clinical insomnia.

The SDI and MSFsc were measured by self-reported responses to the following seven questions: (1) How long do you sleep at night during weekdays? (2) How long do you sleep on the weekend? (3) Considering your own “feeling best performance” rhythms, for how long would you sleep if you were entirely free for the day? (4) What time did you get up on weekdays in the last month? (5) What time did you get up on weekends in the last month? (6) What time did you fall asleep on weekdays in the last month? (7) What time did you fall asleep on weekends in the last month? SDI and MSFsc values were calculated as previously described (22, 27). A higher score for SDI indicated more sleep debt and a higher score for MSFsc indicated a delayed sleep phase.

#### Loneliness

The Three-Item Loneliness Scale (TILS) is a validated three-item self-report questionnaire that assesses loneliness (28, 29). The score for the TILS was summed, with higher scores indicating more severe loneliness.

#### Feelings of School-Refusal

Feelings of school refusal were measured using the Feelings of School-Avoidance Scale (FSAS), “aversion to attending school” (30). The subscale consists of six items, such as “I want to miss school” and “I want to go home as soon as classes are over,” where higher scores indicate stronger aversion to attending school. The alpha coefficient was 0.81, and criterion-related validity was confirmed for social support, self-esteem, and trait anxiety.

#### Model Setting

We hypothesized the following model: COVID-19-related anxiety affects sleep problems, loneliness, and feelings of school refusal; sleep problems affect loneliness and feelings of school refusal; and loneliness affects feelings of school refusal (Figure 1). The model was set with COVID-19-related anxiety, sleep problems, loneliness, and school refusal as latent variables. All latent variables consisted of three observed variables with high factor loads, except for sleep problems. Sleep problems consisted of the AIS, SDI, and MSFsc scores. Error variances were omitted from the model. SEM was performed to confirm the accuracy of the hypothesized model.

### Statistical Analysis

Data were analyzed using SPSS and AMOS Graphics version 26.0 (IBM Inc., Tokyo, Japan). Pearson product-moment correlation analyses were conducted to examine the relationships between the SAVE-6, AIS, SDI, MSFsc, TILS, and FSAS scores. In general, an *r* value of >.1 was taken to indicates a small effect size, a value >.3 to indicate a moderate effect size, and a value >.5 to indicate a large effect size (31).

Structural equation modeling was performed to confirm the accuracy of the hypothesized model. We evaluated the following fit indices: chi-square (χ^2^), goodness-of-fit index (GFI), adjusted goodness-of-fit index (AGFI), comparative fit index (CFI), standardized root mean square residual (SRMR), and root mean square error of approximation (RMSEA). These are absolute fit indices, and it is suggested that these indices should be reported at a minimum (32, 33). A good model fit of χ^2^ provided an insignificant result at a threshold of 0.05. However, when large samples are used, the χ^2^ statistic is essentially a statistical significance test that is sensitive to sample size, meaning that the χ^2^ statistic nearly always rejects the model (32). GFI, AGFI, and CFI can range between 0 and 1, and the closer the values are to 1, the better is the fit of the model. When the SRMR and RMSEA values were ≤ .08, the indices indicated that the model fits well (32, 33).

We used bias-corrected bootstrapped estimates (34) to examine whether sleep problems mediated the relationship between COVID-19-related anxiety and loneliness, and whether loneliness mediated the relationship between sleep problems and school refusal. These estimates are robust to deviations from normality of indirect effects (35, 36). We chose 10,000 bootstrap samples, as recommended in the recent resampling literature, to improve the Monte Carlo accuracy (37). The mediators were significant if the 95% bootstrapped confidence interval (CI) did not include zero.

Finally, hierarchical multiple regression analysis adjusted for age and sex was conducted to determine which sleep problems, insomnia (AIS), sleep debt (SDI), and chronotype (MSFsc) were associated with loneliness and feelings of school refusal. In general, an *R*^2^ value of >.02 was taken to indicates a small effect size, a value >.13 to indicate a moderate effect size, and a value >.26 to indicate a large effect size (31).

## Results

The means and standard deviations of the scores are presented in Table 1. Correlation analysis showed that the TILS was significantly small to moderate correlated (*r*) with the SAVE-6 (*r* = 0.20, *p* < 0.001), SDI (*r* = 0.19, *p* < 0.001), AIS (*r* = 0.33, *p* < 0.01), and FSAS (*r* = 0.49, *p* < 0.001), and that FSAS was significantly small to moderate correlated with SAVE-6 (*r* = 0.16, *p* < 0.001), MSFsc (*r* = −0.22, *p* < 0.001), SDI (*r* = 0.21, *p* < 0.001), and AIS (*r* = 0.43, *p* < 0.001; Table 2).

**Table 1:** Descriptive variables of measures.

	**Mean**	**SD**
SAVE-6	7.26	4.68
MSFsc	3.82	1.61
SDI	1.31	0.83
AIS	4.82	2.69
TILS	1.20	1.50
TSAS	4.64	3.64
Weekday
Sleep-onset-time (h:m)	0:25	1:06
Wake-up-time (h:m)	6:23	0:37
Total sleep time (h)	6.33	0.79
Weekend
Sleep-onset-time (h:m)	0:14	1:17
Wake-up-time (h:m)	7:52	1:33
Total sleep time (h)	7.53	1.21

*AIS, Athens Insomnia Scale; FSAS, Feelings of School Avoidance Scale; MSFsc, sleep-corrected mid-sleep time on free days; SAVE, stress and anxiety to viral epidemics; SD, standardized deviation; SDI, Sleep Debt Index; TILS, Three-Item Loneliness Scale*.

**Table 2:** Correlation between the scales.

	**MSFsc**	**SDI**	**AIS**	**TILS**	**FSAS**
SAVE-6	0.08	−0.03	0.07	0.20**	0.16**
MSFsc		−0.26**	−0.13*	−0.05	−0.22**
SDI			0.27**	0.19**	0.21**
AIS				0.33**	0.43**
TILS					0.49**

*AIS, Athens Insomnia Scale; FSAS, Feelings of School Avoidance Scale; MSFsc, sleep-corrected mid-sleep time on free days; SAVE, stress and anxiety to viral epidemics; SDI, Sleep Debt Index; TILS, Three-Item Loneliness Scale.*

*p <0.05.

**p <0.01.

**Figure 2:**
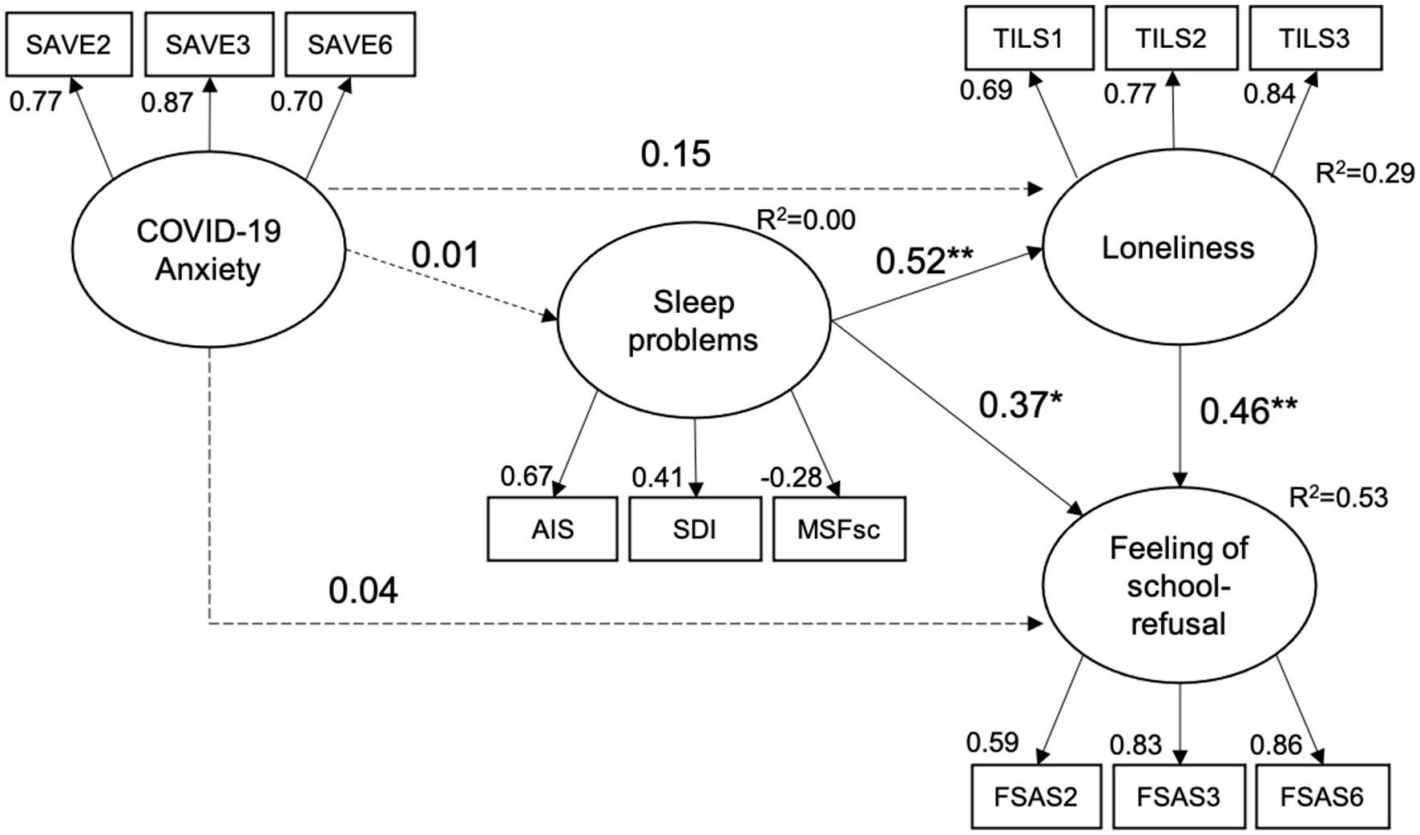
Result of structural equation modeling. Solid lines indicate significant pathways. Dashed lines indicate non-significant pathways. *p < 0.05, **p < 0.01.

The result of SEM showed that the hypothesized model had a relatively good fit ($\chi_{48}^{2}$ = 104.146, *p* < 0.001, GFI = 0.937, AGFI = 0.897, CFI = 0.945, SRMR = 0.053, RMSEA = 0.068; see Figure 2). The results also showed that sleep problems affected loneliness (β = 0.52, 95% CI: 0.34 to 0.70, *p* < 0.001) and feelings of school refusal (β = 0.37, 95% CI: 0.15 to 0.64, *p* < 0.01), and loneliness affected feelings of school refusal (β = 0.47, 95% CI: 0.19 to 0.62, *p* < 0.01; Figure 2).

There was no significant pathway for COVID-19-related anxiety affecting sleep problems (β = 0.01, 95% CI: −0.20 to 0.20), loneliness (β = 0.15, 95% CI: −0.004 to 0.31), or feelings of school refusal (β = 0.04, 95% CI: −0.01 to 0.19). The indirect effects of sleep problems on feelings of school refusal due to loneliness were significant (95% CI: 0.13 to 0.38).

The results of hierarchical multiple regression analysis showed that AIS (β = 0.30, *p* < 0.001) and SDI (β = 0.13, *p* = 0.08) were associated with TILS (*R*^2^ = 0.18, *p* < 0.001), whereas AIS (β = 0.26, *p* < 0.001) and MSFsc (β = −0.14, *p* = 0.01) were associated with FSAS (*R*^2^ = 0.24, *p* < 0.001; Table 3).

**Table 3:** Results of hierarchical multiple regression analysis.

	**Loneliness**			**Feelings of school-refusal**		
	**β**	***t*-Value**	***p*-Value**	**β**	***t*-Value**	***p*-Value**
MSFsc	0.02	0.00	n.s.	−0.14	−2.51	0.01
SDI	0.13	1.74	0.08	0.02	0.35	n.s.
AIS	0.30	4.74	<0.001	0.26	4.64	<0.001
*R* ^2^	0.18		<0.001	0.24		<0.001

*AIS, Athens Insomnia Scale; MSFsc, sleep-corrected mid-sleep time on free days; SDI, Sleep Debt Index. n.s., not significant.*

*Adjusted for age and sex*.

## Discussion

The purpose of this study was to examine the following process model using the SEM: COVID-19-related anxiety affects sleep problems, loneliness, and feelings of school refusal; sleep problems affect loneliness and feelings of school refusal; and loneliness affects feelings of school refusal.

There were significant correlations between the scales for COVID-19-related anxiety, sleep problems, loneliness, and feelings of school refusal. These findings were consistent with those of previous studies (9–14). It has been suggested that sleep problems are associated with aversion to loneliness and with feelings of school refusal. On the other hands, COVID-19-related anxiety was not correlated with sleep debt and insomnia as well as chronotype. Previous studies reported both sleep debt and insomnia were associated with anxiety symptoms (20, 21). The COVID-19-related anxiety and traditional anxiety symptoms (e.g., state–trait anxiety) might have different status.

Structural equation modeling showed that the hypothesized model fit well. Unlike previous studies (14, 18), this study revealed that COVID-19-related anxiety did not affect sleep problems, loneliness, or school refusal. The participants in previous studies (14, 18) were from the general population (age range: 13–90 years) or healthcare workers, including medical institutions designated for treating patients with COVID-19. Therefore, sleep in adolescents may not be aggravated by COVID-19-related anxiety. In addition, a previous report that suggested that students in Japan avoided going to school due to COVID-19-related anxiety (6), was conducted early during the COVID-19 pandemic. The present study was conducted approximately 19 months after the first reported case of COVID-19 in January 2020. This implies that the association between COVID-19-related anxiety, sleep, loneliness, and school refusal in adolescents may have changed during the course of the pandemic.

No previous study has shown that sleep problems affect school refusal by both a direct and an indirect pathway through exacerbation of loneliness. Maeda et al. (11) showed that a sleep education program for primary school students successfully achieved a more routine nighttime sleep pattern and a regular life rhythm, which prevented school refusal during subsequent junior high school years. Therefore, sleep-enhancing interventions, such as sleep education, may be effective in preventing loneliness and school refusal among high school students with sleep problems. In addition, among sleep-related factors, insomnia commonly affected loneliness and school refusal. On the other hand, sleep debt is likely to affect loneliness only, while chronotype affects school refusal only. In this light, as prevention of school refusal in adolescents, an indirect pathway approach *via* loneliness could be effective in improving insomnia and sleep debt, while a direct pathway approach could be effective in improving insomnia and chronotype.

Recently, cognitive behavior therapy for insomnia (CBT-I) has been effective for adolescents or young adults with insomnia (38, 39), and CBT-I plus bright-light therapy has been effective for adolescents with a chronotype of delayed sleep-wake phase (40). However, to the best of our knowledge, no effective CBT approach has been developed to improve sleep debt. Besides sleep problems, perceived social support has also been associated with loneliness (41). To prevent loneliness and school refusal, a comprehensive CBT approach targeting sleep problems and social support should be developed.

This study had some limitations. First, this cross-sectional study could not identify a causal relationship among COVID-19-related anxiety, sleep problems, loneliness, and school refusal. To clarify this, future prospective follow-up studies are needed to evaluate the influences of COVID-19-related anxiety and sleep problems on longitudinal changes in loneliness and school refusal. The participants in this study were from a public senior high school. To ensure generalizability, future research should be conducted on senior high school students nationwide. Finally, we measured the symptoms solely using self-reported scales and limited sociodemographic data. Using objective sleep measures and other sociodemographic data such as intelligence quotient or academic achievement, social isolation, family history, or economic status in future studies may help to illuminate specific sleep variables that contribute to worsening loneliness and school refusal.

## Conclusion

The findings of this study revealed that sleep-related factors, such as insomnia, sleep debt, and chronotype, may trigger loneliness and school refusal among adolescents. Although the present study was conducted on senior high school students, it is necessary to examine whether this model, with direct and indirect pathways, can also be applied to elementary and junior high school students.

## Data Availability Statement

The raw data supporting the conclusions of this article will be made available by the authors, without undue reservation.

## Ethics Statement

The studies involving human participants were reviewed and approved by the Ethics Committee of Tokyo Kasei University (ID: Ita-E2021-15). Written informed consent from the participants' legal guardian/next of kin was not required to participate in this study in accordance with the national legislation and the institutional requirements.

## Author Contributions

IO: conceptualization, funding acquisition, methodology, project administration, and roles/writing—original draft. YH: conceptualization and investigation. OS, YK, and YT: investigation and project administration. All authors contributed to the article and approved the submitted version.

## Funding

This work was partially supported by a Grant-in-Aid for Scientific Research (C) (KAKENHI) awarded by the Japan Society for the Promotion of Science (JSPS; Grant Number 19K03348).

## Conflict of Interest

IO received grants from NEC Solution Innovators Co., Ltd., and Infocom Co.; lecture fees from Otsuka Pharmaceutical Co., Ltd., MSD K.K., Eisai Co., Ltd.; and consultation fees from NEC Solution Innovators Co., Ltd. and Suntory Wellness Ltd. for projects unrelated to the submitted work. OS is employed by BiosPyxis Co., Ltd. YK was employed by Medimpl Corporation at the time of the study, and is employed by FiveVai, Inc. at time of publication. The remaining authors declare that the research was conducted in the absence of any commercial or financial relationships that could be construed as a potential conflict of interest.

## Publisher's Note

All claims expressed in this article are solely those of the authors and do not necessarily represent those of their affiliated organizations, or those of the publisher, the editors and the reviewers. Any product that may be evaluated in this article, or claim that may be made by its manufacturer, is not guaranteed or endorsed by the publisher.
